# Prevalence and predictive factors of nocturia in patients with obstructive sleep apnea syndrome: A retrospective cross-sectional study

**DOI:** 10.1371/journal.pone.0267441

**Published:** 2022-04-27

**Authors:** Yeon Hak Chung, Jae Rim Kim, Su Jung Choi, Eun Yeon Joo

**Affiliations:** 1 Department of Neurology, Neuroscience Center, Samsung Medical Center, Sungkyunkwan University School of Medicine, Seoul, Korea; 2 Graduate School of Clinical Nursing Science, Sungkyunkwan University, Seoul, Korea; Sapienza University of Rome, ITALY

## Abstract

**Objectives:**

Many patients with obstructive sleep apnea syndrome (OSAS) have nocturia. However, the predictive index of nocturia in patients with OSAS is currently not well known. We aimed to investigate the prevalence of nocturia in patients with OSAS and determine the factors that could predict nocturia in these patients.

**Methods:**

In this retrospective cross-sectional study, we enrolled 1,264 untreated patients with OSAS (Apnea-Hypopnea Index, AHI ≥5/h on polysomnography [PSG]) from January 2017 to January 2020. Participants completed the Beck Depression Inventory-II (BDI-II), Pittsburgh Sleep Quality Index (PSQI), Insomnia Severity Index (ISI), and Epworth Sleepiness Scale. Participants were divided by sex and then subdivided into nocturia and non-nocturia groups according to the following question, “Do you go to the bathroom two times or more during your sleep?” Participants’ characteristics and underlying disease were investigated, and all information, including PSG data, was compared between the two groups using the t-test or chi-square test.

**Results:**

Overall, 35.2% (337/958) of male participants with OSAS and 59.8% (183/306) of female participants with OSAS had nocturia. The nocturia group was older; scored higher on the BDI-II, PSQI, and ISI; and had more underlying disease in both sexes. There was no difference in the AHI between the two groups among both sexes, but the hypoxia-related PSG parameters and sleep quality parameters, such as higher 90% oxygen desaturation index (90% ODI), lesser N3 sleep, and higher wakefulness after sleep onset, were worse among male participants with OSAS in the nocturia group than in the non-nocturia group. In multivariate logistic analysis, 90% ODI was an independent risk factor associated with nocturia in male participants with OSAS.

**Conclusions:**

Considerable number of patients with OSAS had nocturia and poor sleep quality. Nocturia should be evaluated in male OSAS patients with severe hypoxia observed during sleep.

## Introduction

Nocturia is defined as an instance of waking up to urinate one or more times during sleep [1]. In clinical practice, nocturia refers to two or more nighttime urinations, because urinating twice or more during sleep results in nocturia that affects well-being and health [2]. Nocturia is extremely prevalent that 28.5% of the total population has it [3]. Nocturia increases with age in both sexes [3]. Overall, 16.5% of those younger than 50 years of age and 60% of those older than 70 years of age complain of nocturia [3]. In the population younger than 50 years of age, nocturia is more common in women than in men, and in the population older than 50 years, the frequency of nocturia in men increases sharply [4]. Nocturia is common in both sexes, although the distribution of its incidence according to age is different [4, 5].

Since nocturia is one of the lower urinary tract symptoms, many causes and mechanisms of nocturia, such as polyuria, nocturnal polyuria, reduced bladder capacity, primary sleep disorder, or psychiatric disorder, have been suggested [6]. In addition, urological diseases, such as prostate disease [3, 7], cardiovascular disease [8], condition requiring diuretics [8], kidney disease [7], neurodegenerative disease such as Parkinson disease [9], or diabetes [3], may cause nocturia. A substantial number of patients complaining of sleep maintenance disorders caused by nocturia are accompanied by obstructive sleep apnea syndrome (OSAS), and about 50% of patients with OSAS have nocturia [10]. There are reports of reduced episodes of nocturia after applying positive airway pressure treatment to patients with OSAS [11], suggesting that OSAS is main cause of nocturia.

There are several mechanisms that physiologically explain the cause of nocturia in OSAS. If negative pressure is continuously maintained in the thoracic cavity due to respiratory disturbance during sleep, venous blood increases to the heart, increasing the secretion of the atrial natriuretic peptide in plasma and urine and suppressing the secretion of the anti-diuretic hormone, inhibiting urine concentration and causing nocturnal polyuria [12]. Additionally, there is a theory that periodic hypoxia caused by OSAS causes instability in the bladder and leads to nocturia [13], indicating that OSAS and nocturia are very closely related. However, among the various sleep-related parameters caused by OSAS, the factor that can predict nocturia has not yet been revealed.

We hypothesized that nocturia in patients with OSAS would be related to OSAS and the severity of OSAS. Moreover, we hypothesized that among the polysomnography (PSG) parameters, hypoxia-related parameters in particular could predict nocturia in patients with OSAS. By analyzing the comorbid disease of nocturia and results of PSG, we aimed to investigate the prevalence of nocturia in patients with OSAS and to determine relevant sleep parameters and clinical factors that can predict nocturia in patients with OSAS.

## Material and methods

### Ethics statement

The research standards, methods, and evaluations used in this study were reviewed, and an exemption of consent was approved by the Institutional Review Board of the Samsung Medical Center because of the retrospective study design (approval number: 2020-04-049).

### Study design and participants

In this retrospective cross-sectional study, we used the medical records of patients who underwent PSG at the sleep center of Samsung Medical Center from January 2017 to January 2020. All participants were older than 18 years of age, completed all the standardized questionnaires, and were diagnosed with OSAS and had an Apnea-Hypopnea Index (AHI) of 5 or more per hour confirmed by PSG with daytime symptoms, such as daytime sleepiness and fatigue, or sleep apnea or snoring during sleep [14]. Patients who were younger than 18 year of age and did not complete the questionnaires or had incomplete medical records were excluded. Patients with prostate disease and bladder disease, which may present with symptoms of lower urinary tract symptoms including nocturia were also excluded.

It was assumed that there would be differences in the mechanisms and risk factors for nocturia in men and women. Therefore, men and women were separated. Those who gave a positive answer to the question, “Do you go to the bathroom two times or more during your sleep?” were categorized into the nocturia group, whereas those who gave a negative answer were categorized into the non-nocturia group. Participants were asked to respond in consideration of the last month.

### Questionnaires

Participants completed the Korean version of the Pittsburgh Sleep Quality Index-K (PSQI-K) [15], Insomnia Severity Index (ISI) [16], Epworth Sleepiness Scale [17], and Korean Beck Depression Inventory-2 (K-BDI-II) [18]. In addition, general characteristics including age, body mass index, lifestyle (smoking frequency, alcohol intake, caffeine intake), and comorbidities (hypertension, diabetes, dyslipidemia, cardiovascular disease, Parkinson disease, stroke, and chronic kidney disease), which were known to be related to nocturia, were investigated [3, 7, 8].

### Polysomnography

Sleep tests were performed using the Embla N7000 (Medcare-Embla^®^, Reykjavik, Iceland). Test items included six channels of electroencephalography (EEG) (C3-A2, C4-A1, F3-A2, F4-A1, O3-A2, and O2-A1), four channels of electro-oculography, and one channel of chin electromyography (chin EMG) to measure the sleep phase and arousal frequency. For the respiration index, nasal air pressure was measured using a pressure sensor. Respiratory movement was measured using the thoracic and abdominal belts to assess the respiratory effort. Oximetry was used to measure oxygen saturation in the index finger, and snoring intensity was measured with an auditory sensor. To evaluate leg movement, we used two-channel EMG of the tibialis anterior muscle. Behavioral disturbances and postures during sleep were recorded by recording a video while simultaneously measuring the electrocardiogram parameters and checking the sleep posture via a position sensor.

The PSG evaluation was scored according to the American Academy of Sleep Medicine Manual [19]. The sleep structure was analyzed through EEG, EMG, and electro-oculography, and the ratio of the non-rapid eye movement sleep stage 1 (N1), non-rapid eye movement sleep stage 2 (N2), non-rapid eye movement sleep stage 3 (N3), and rapid eye movement (REM) sleep stages was investigated. Sleep patterns were measured by sleep latency, wakefulness after sleep onset (WASO), total sleep time (TST), sleep efficiency, and arousal index. Apnea is a condition in which breathing stops for >10 seconds, which is measured by a decrease of airflow >90% through the temperature sensor of the nasal cavity. Hypopnea is a condition in which airflow decreases by 30% or more for 10 seconds or more with oxygen saturation decreased by 4% or more of its baseline, or with EEG arousal observed [19]. OSAS was defined when the AHI was 5 or more per hour with daytime symptoms, such as daytime sleepiness and fatigue, or sleep apnea or snoring during sleep [14].

### Statistical analysis

Participants’ characteristics and PSG results are described as mean and standard deviation or frequency and percentage. The t-test or chi-square test was used to evaluate differences between the nocturia and non-nocturia groups. To identify nocturia-related sleep parameters, univariate logistic regression analysis was performed for variables that showed significant differences in the comparative test and variables found to have significant differences in previous studies. Multivariate logistic regression analysis was performed for variables that were significant at a *p*-value of 0.10 in univariate logistic regression analysis. For all statistical analyses, SPSS 25.0 (IBM Corp, Armonk, NY, USA) was used, and when the *p*-value was <0.05, it was determined to be statistically significant.

## Results

### Participant characteristics

**Fig 1:**
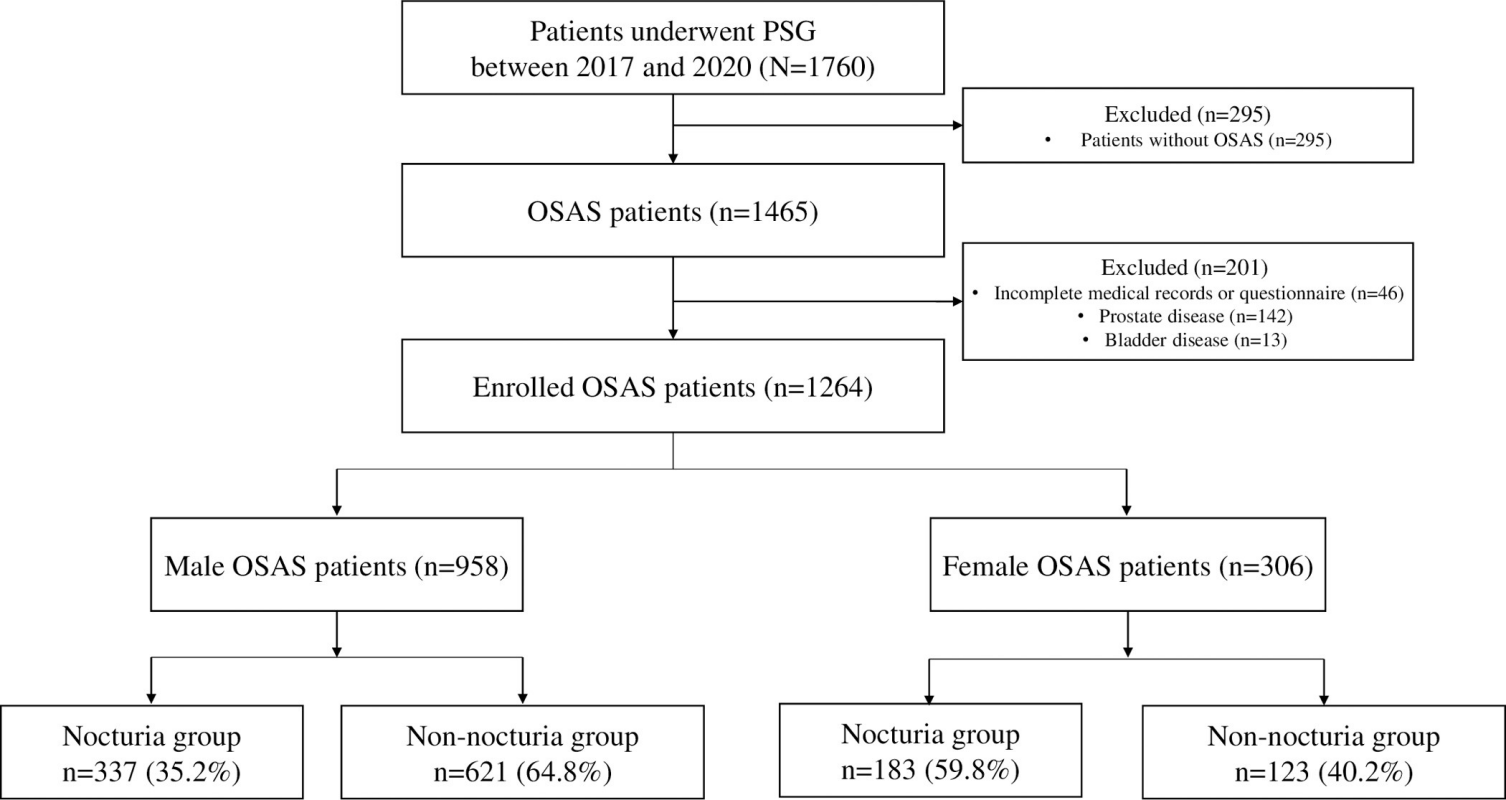
Study flow chart. We screened 1,465 patients with OSAS (Apnea-Hypopnea Index≥5/h on PSG) out of 1760 patients who underwent PSG. Patients who had incomplete medical records or questionnaires, prostate disease, or bladder disease were excluded. We registered 1,264 participants, and they were divided into male and female groups and then subdivided into the nocturia and non-nocturia groups according to the following question, “Do you go to the bathroom two times or more during your sleep?”. OSAS: obstructive sleep apnea syndrome. PSG: polysomnography.

In total, 1,760 patients underwent PSG during the study period, of which 1,465 were diagnosed with OSAS. Among them, 1,264 patients were selected as final study participants, excluding 46 who did not complete the standardized questionnaires or had incomplete medical records, 142 who had prostate disease, and 13 who had bladder disease (Fig 1). Ultimately, 1,264 participants were analyzed, and there were 958 male participants with OSAS and 306 female participants with OSAS. Among male participants with OSAS, 337 (35.2%) were classified in the nocturia group, and among female participants with OSAS, 183 (59.8%) were classified in the nocturia group.

Participants’ mean age was higher in the nocturia group than in the non-nocturia group among both sexes (men: 55.1±13.3 versus [vs.] 49.6±13.7, *p*<0.001; women: 61.6±11.4 vs. 56.1±13.2, *p*<0.001). There were no differences in alcohol consumption, smoking frequency, caffeine intake, and body mass index of both sexes between the two groups. Among male participants with OSAS in the nocturia group, there were significant higher incidences of hypertension, diabetes, dyslipidemia, use of diuretics, cardiovascular disease, stroke, and chronic kidney disease than among those in the non-nocturia group. There were no differences in those diseases among female participants with OSAS between the two groups. Compared to the non-nocturia group, the nocturia group rated subjective sleep quality worse (PSQI-K, men: 8.0±3.7 vs. 6.8±5.1, *p*<0.001; women: 9.7±4.4 vs. 8.5±4.0, *p* = 0.013) and complained of more severe insomnia-related symptoms (ISI, men: 11.8±5.7 vs. 9.1±6.9, *p*<0.001; women: 13.9±7.1 vs. 11.8±6.3, *p* = 0.011) in both sexes. Depression was also higher among male participants with OSAS in the nocturia group than among those of the non-nocturia group, but there was no difference in depression among female participants with OSAS between the groups (K-BDI-II, men: 13.9±8.9 vs. 10.6±7.5, *p*<0.001; women (nocturia and non-nocturia groups): 17.2±9.4 and 15.8±9.1, respectively; *p* = 0.172) (Table 1).

**Table 1:** Participants’ characteristics by group and sex (N = 1,264).

Characteristic	Men (n = 958)			Women (n = 306)		
	Nocturia (n = 337)	Non-nocturia (n = 621)	*p-*value	Nocturia (n = 183)	Non-nocturia (n = 123)	*p-*value
Age (years)	55.1±13.3	47.3±13.1	<0.001	61.6±11.4	56.1±13.2	<0.001
Smoking frequency (pack-year)	5.0±11.0	4.0±8.3	0.223	0.2±1.4	0.0±0.0	0.070
Alcohol intake (g/week)	113.0±272.6	116.7±342.0	0.877	8.0±26.5	7.9±26.6	0.981
Caffeine intake (cups/day)	1.9±1.5	1.8±1.3	0.372	1.0±1.0	1.1±1.1	0.556
Body mass index (kg/m^2^)	27.0±4.4	27.0±3.9	0.980	25.7±4.6	25.1±5.5	0.296
Hypertension	163 (48.4%)	221 (35.6%)	<0.001	80 (43.7%)	41 (33.3%)	0.069
Diabetes mellitus	72 (21.4%)	60 (9.7%)	<0.001	35 (19.1%)	17 (13.8%)	0.226
Dyslipidemia	110 (32.6%)	160 (25.8%)	0.024	76 (41.5%)	42 (34.1%)	0.193
Use of diuretics	16 (4.7%)	6 (1.0%)	<0.001	3 (1.6%)	2 (1.6%)	1.000
Parkinson disease	1 (0.3%)	2 (0.3%)	1.000	5 (2.7%)	1 (0.8%)	0.407
Cardiovascular disease	44 (13.1%)	20 (3.2%)	<0.001	9 (4.9%)	1 (0.8%)	0.054
Stroke	19 (5.6%)	16 (2.6%)	0.016	12 (6.6%)	5 (4.1%)	0.351
Chronic kidney disease	16 (4.7%)	10 (1.6%)	0.004	1 (0.5%)	1 (0.8%)	1.000
PSQI-K	8.0±3.7	6.8±5.1	<0.001	9.7±4.4	8.5±4.0	0.013
ISI	11.8±5.7	9.1±6.9	<0.001	13.9±7.1	11.8±6.3	0.011
ESS	9.8±5.4	9.6±4.5	0.639	8.1±5.3	8.7±5.2	0.292
K-BDI-II	13.9±8.9	10.6±7.5	<0.001	17.2±9.4	15.8±9.1	0.172

Values are expressed as mean±standard deviation or number (percentage).

PSQI-K: Korean version of the Pittsburg Sleep Quality Index, ISI: Insomnia Severity Index, ESS: Epworth Sleep Scale, K-BDI-II: Korean Beck Depression Inventory-II.

### Sleep parameters

The percentage of WASO was higher (men: 18.1±12.6 vs. 13.9±10.1, *p*<0.001; women: 17.0±10.7 vs. 13.9±10.8, *p* = 0.015) and sleep efficiency was significantly lower (men: 79.9±13.3 vs. 84.1±10.9, *p*<0.001; women: 80.1±11.3 vs. 83.0±12.7, *p* = 0.043) in the nocturia group than in the non-nocturia group among both sexes. The TST was shorter (men: 340.1±70.1 vs. 358.0±61.2, *p*<0.001) only in male participants in the nocturia group than in the non-nocturia group. Regarding the sleep stage the N1 sleep% was longer (men: 26.9±15.0 vs. 23.6±13.1, *p =* 0.001), N3 sleep% was shorter (men: 2.8±4.8 vs. 4.6±6.3, *p*<0.001), REM sleep% was shorter (men: 17.6±7.2 vs. 18.7±6.5, *p =* 0.013) in the nocturia group than in the non-nocturia group among only male participants. There was no difference in the AHI between the groups, but the arousal index (men: 32.0±17.9 vs. 29.1±15.8, *p =* 0.011) and hypoxia-related index such as the 90% oxygen desaturation index (90% ODI) (men: 13.6±22.1 vs. 10.3±17.7, *p =* 0.018) were higher in the nocturia group than in the non-nocturia group among male participants (Table 2).

**Table 2:** Polysomnography parameters by group and sex (N = 1,264).

Variable	Men (n = 958)			Women (n = 306)		
	Nocturia (n = 337)	Non-Nocturia (n = 621)	*p*-value	Nocturia (n = 183)	Non-Nocturia (n = 123)	*p*-value
Total sleep time, min	340.1±70.1	358.0±61.2	<0.001	357.8±63.9	366.0±66.5	0.279
Sleep latency, min	10.7±16.6	10.3±16.9	0.725	15.9±20.5	18.0±30.3	0.461
REM sleep latency, min	104.1±70.7	105.4±60.9	0.764	124.6±69.9	115.2±70.2	0.250
Sleep efficiency, %	18.1±12.6	13.9±10.1	<0.001	80.1±11.3	83.0±12.7	0.043
WASO, %	79.9±13.3	84.1±10.9	<0.001	17.0±10.7	13.9±10.8	0.015
Sleep stage, %						
N1 sleep	26.9±15.0	23.6±13.1	0.001	19.1±11.5	17.8±11.5	0.331
N2 sleep	52.7±13.3	53.1±11.4	0.627	56.9±11.5	56.6±10.8	0.819
N3 sleep	2.8±4.8	4.6±6.3	<0.001	5.0±6.8	6.2±7.0	0.134
REM sleep	17.6±7.2	18.7±6.5	0.013	19.0±6.9	19.4±6.9	0.621
Arousal index, events/h	32.0±17.9	29.1±15.8	0.011	22.8±13.3	21.7±12.1	0.448
AHI, events/h	36.5±24.7	33.9±17.6	0.112	25.2±20.9	23.7±23.4	0.565
Apnea index	17.4±22.8	14.7±19.9	0.071	7.0±14.3	5.8±11.6	0.462
Hypopnea index	19.1±13.0	19.2±11.9	0.870	18.2±12.6	17.9±16.9	0.844
Lowest SaO2, %	81.8±8.4	82.1±8.2	0.576	84.3±7.5	84.9±6.4	0.494
ODI, events/h	30.7±24.6	28.6±1.3	0.175	21.1±20.6	20.9±1.5	0.935
90% ODI, events/h	13.6±22.1	10.3±17.7	0.018	5.8±16.3	6.7±19.4	0.679

Values are expressed as mean±standard deviation.

REM: rapid eye movement, WASO: wakefulness after sleep onset, N1: non-rapid eye movement sleep stage 1, N2: non-rapid eye movement sleep stage 2, N3: non-rapid eye movement sleep stage 3, AHI: Apnea-Hypopnea Index, SaO_2_: oxygen saturation, ODI: oxygen desaturation index (the number of 3% oxygen desaturations per hour of estimated sleep time), 90% ODI: 90% oxygen desaturation index (the number of 3% oxygen desaturations below 90% per hour of estimated sleep time).

### Predictor of nocturia

Factors that showed a significant difference between the nocturia and non-nocturia groups were identified, and univariate logistic regression analysis was performed for those factors and factors known to have significant differences in previous studies. Age, sex, hypertension, diabetes mellitus, dyslipidemia, use of diuretics, Parkinson disease, cardiovascular disease, stroke, chronic kidney disease, and PSG parameters were included as variables. The PSQI-K, ISI, and K-BDI-II values, which showed a significant difference between the two groups, were not included in the regression analysis as they were assumed to be results rather than cause of nocturia. In univariate logistic regression analysis of male participants with OSAS, age, hypertension, diabetes, dyslipidemia, use of diuretics, cardiovascular disease, stroke, and chronic kidney disease were significant clinical factors. Among PSG parameters, the TST, sleep efficiency, WASO, N1 sleep, N3 sleep, REM sleep, arousal index, and 90% ODI showed significant differences between the groups. Multivariate logistic regression analysis of male participants was performed for variables shown to be significant variables in univariate logistic regression analysis (*p*-value >0.10). Age (odd ratio = 1.036, 95% confidence interval: 1.022–1.051), diabetes mellitus (1.847, 1.127–2.805), cardiovascular disease (2.658, 1.442–4.902), and 90% ODI (1.020, 1.005–1.036) were identified as independent risk factors (Table 3).

**Table 3:** Results of logistic regression analysis of factors related to nocturia in male participants with obstructive sleep apnea syndrome (N = 958).

Risk factor	Univariate regression analysis		Multivariate regression analysis	
	Odds ratio (95% CI)	*p*-value	Odds ratio (95% CI)	*p*-value
**Clinical Factor**				
Age	1.046 (1.034–1.057)	<0.001	1.036 (1.022–1.051)	<0.001
Hypertension	1.696 (1.295–2.220)	<0.001	0.953 (0.692–1.311)	0.767
Diabetes mellitus	2.540 (1.750–3.687)	<0.001	1.847 (1.217–2.805)	0.004
Dyslipidemia	1.396 (1.045–1.866)	0.024	0.741 (0.525–1.048)	0.090
Use of diuretics	5.109 (1.980–13.183)	0.001	2.525 (0.824–7.735)	0.105
Parkinson disease	0.921 (0.083–10.196)	0.947		
Cardiovascular disease	4.513 (2.612–7.796)	<0.001	2.658 (1.442–4.902)	0.002
Stroke	2.259 (1.146–4.454)	0.019	1.170 (0.533–2.568)	0.696
Chronic kidney disease	3.045 (1.366–6.788)	0.006	1.662 (0.652–4.237)	0.287
**Polysomnography parameter**				
Total sleep time, min	0.996 (0.994–0.998)	<0.001	1.003 (0.999–1.007)	0.189
Sleep latency, min	1.001 (0.994–1.009)	0.725		
REM sleep latency, min	1.000 (0.998–1.002)	0.753		
Sleep efficiency, %	0.972 (0.961–0.983)	<0.001	1.006 (0.950–1.066)	0.833
WASO, %	1.033 (1.021–1.045)	<0.001	1.037 (0.978–1.100)	0.227
Sleep stage, %				
N1 sleep	1.017 (1.008–1.027)	<0.001	1.000 (0.981–1.020)	0.979
N2 sleep	0.997 (0.986–1.008)	0.610		
N3 sleep	0.943 (0.919–0.968)	<0.001	0.991 (0.961–1.021)	0.544
REM sleep	0.975 (0.956–0.995)	0.013	0.997 (0.973–1.021)	0.807
Arousal index, events/h	1.011 (1.003–1.019)	0.009	0.991 (0.972–1.012)	0.406
AHI, events/h	1.005 (0.999–1.010)	0.112		
Apnea index	1.006 (1.000–1.012)	0.060	0.996 (0.982–1.010)	0.581
Hypopnea index	0.999 (0.988–1.010)	0.870		
Lowest SaO2, %	0.995 (0.980–1.012)	0.575		
ODI, events/h	1.004 (0.998–1.010)	0.175		
90% ODI, events/h	1.009 (1.002–1.015)	0.012	1.020 (1.005–1.036)	0.011

REM: rapid eye movement, WASO: wakefulness after sleep onset, N1: non-rapid eye movement sleep stage 1, N2: non-rapid eye movement sleep stage 2, N3: non-rapid eye movement sleep stage 3, AHI: Apnea-Hypopnea Index, SaO_2_: oxygen saturation, 90% ODI: oxygen desaturation index (the number of 3% oxygen desaturations per hour of estimated sleep time), 90% ODI: 90% oxygen desaturation index (the number of 3% oxygen desaturations below 90% per hour of estimated sleep time), CI: confidence interval.

In univariate logistic regression analysis of female participants, age was a significant clinical factor and sleep efficiency and WASO were significant PSG parameters. Multiple logistic regression analysis of female participants was performed for variables shown to be significant variables in univariate logistic regression analysis (*p*-value >0.10). Age (odds ratio = 1.030, 95% confidence interval: 1.009–1.051) was identified as an independent risk factor (Table 4).

**Table 4:** Results of logistic regression analysis of factors related to nocturia in female participants with obstructive sleep apnea syndrome (N = 306).

Risk factor	Univariate regression analysis		Multivariate regression analysis	
	Odds ratio (95% CI)	*p*-value	Odds ratio (95% CI)	*p*-value
**Clinical Factor**				
Age	1.037 (1.017–1.058)	<0.001	1.030 (1.009–1.051)	0.005
Hypertension	1.553 (0.966–2.498)	0.069	1.191 (0.718–1.974)	0.499
Diabetes mellitus	1.475 (0.785–2.771)	0.228		
Dyslipidemia	1.370 (0.852–2.202)	0.194		
Use of diuretics	1.008 (0.166–6.124)	0.993		
Parkinson disease	3.427 (0.395–29.697)	0.264		
Cardiovascular disease	6.310 (0.789–50.456)	0.082	4.017 (0.492–32.801)	0.194
Stroke	1.656 (0.568–4.825)	0.355		
Chronic kidney disease	0.670 (0.042–10.819)	0.778		
**Polysomnography parameter**				
Total sleep time, min	0.998 (0.994–1.002)	0.278		
Sleep latency, min	0.997 (0.988–1.006)	0.463		
REM sleep latency, min	1.002 (0.999–1.005)	0.250		
Sleep efficiency, %	0.979 (0.959–1.000)	0.045	1.022 (0.961–1.086)	0.492
WASO, %	1.029 (1.005–1.054)	0.017	1.044 (0.974–1.118)	0.222
Sleep stage, %				
N1 sleep	1.010 (0.990–1.031)	0.332		
N2 sleep	1.002 (0.982–1.023)	0.818		
N3 sleep	0.975 (0.944–1.008)	0.136		
REM sleep	0.992 (0.959–1.025)	0.620		
Arousal index, events/h	1.007 (0.989–1.026)	0.448		
AHI, events/h	1.003 (0.993–1.014)	0.564		
Apnea index	1.007 (0.989–1.026)	0.464		
Hypopnea index	1.002 (0.986–1.018)	0.844		
Lowest SaO2, %	0.988 (0.956–1.022)	0.494		
ODI, events/h	1.000 (0.990–1.011)	0.935		
90% ODI, events/h	0.997 (0.985–1.010)	0.679		

REM: rapid eye movement, WASO: wakefulness after sleep onset, N1: non-rapid eye movement sleep stage 1, N2: non-rapid eye movement sleep stage 2, N3: non-rapid eye movement sleep stage 3, AHI: Apnea-Hypopnea Index, SaO_2_: oxygen saturation, ODI: oxygen desaturation index (the number of 3% oxygen desaturations per hour of estimated sleep time), 90% ODI: 90% oxygen desaturation index (the number of 3% oxygen desaturations below 90% per hour of estimated sleep time), CI: confidence interval.

## Discussion

Although the close relationship between OSAS and nocturia is well known [10, 20], the predictive index of nocturia among PSG parameters is still not well established. Since there are many underlying diseases or drugs that cause nocturia, various clinical information and considerations are needed to analyze the effects of OSAS itself on nocturia [5]. Questionnaires and participants’ medical records were checked to identify participants’ medications and underlying diseases. Since the pathophysiology of nocturia and OSAS showed differences according to sex, the two groups were divided and compared [4, 21, 22]. In this study, the prevalence of nocturia due to OSAS was obtained for the first time in a large population in an Asian country, and it was confirmed that the hypoxia-related sleep parameter is an independent risk factor for nocturia in male participants with OSAS.

In male participants with OSAS, there were significant differences in underlying diseases such as hypertension, diabetes mellitus, dyslipidemia, conditions requiring use of diuretics, cardiovascular disease, stroke, and chronic kidney disease between the nocturia group and non-nocturia groups. Also, substantial sleep parameters (TST, WASO, sleep efficiency, N1 sleep, N3 sleep, REM sleep, and arousal index) as well as 90% ODI were different between the two groups. Among those aforementioned factors, 90% ODI was found to be an independent risk factor in multiple logistic regression analysis. The severity of OSAS is determined by the AHI score [14], and there are some studies on the relationship between OSAS and AHI [23, 24]. However, in the present study, there was no significant difference according to the presence of nocturia and the degree of AHI. Additionally, the oxygen desaturation index (ODI) value was insignificant, and the 90% ODI value was significant. It can be considered that nocturia in patients with OSAS is related to moderate intermittent hypoxia represented by intermittent desaturation below 90%, because the ODI includes a minimal desaturation event at an oxygen saturation above 90%. Endeshaw et al. showed similar results that there was no significant difference in the ODI between the study groups, but there was a significant difference in ODI events occurring with oxygen saturation below 90% [23]. Considering the above results, nocturia may be related to hypoxia as a result of OSAS rather than OSAS itself causing nocturia, which would negate the previous study hypothesis that OSAS would be related to OSAS severity represented as AHI but confirm this study hypothesis that PSG parameters, hypoxia-related parameters in particular could predict nocturia in patients with OSAS.

In female participants with OSAS, age was significantly different between the two groups and the PSG parameters WASO and sleep efficiency were significantly different. Age was the only independent variable in multiple regression analysis. Unlike male participants with OSAS, female participants with OSAS had no hypoxia-related PSG parameters associated with nocturia. It is noted that apnea index of female participants was significantly lower (6.5±13.3/h) than hypopnea index (18.1±14.5/h), while apnea index (15.7±21.0/h) of male participants was not different to hypopnea index (19.2±12.3/h). Hypopnea index includes flow reduction with arousal or desaturation in definition [19]. It was not usual to differentiate hypopnea with arousals without definite oxygen saturation from participants’ hypopnea index. However, this finding may enable to assume that female OSAS participants had less hypoxia-related index than male OSAS participants. It also supports the role of hypoxia-related parameters on nocturia in patients with OSAS.

There was a difference between the nocturia and non-nocturia groups of male participants with OSAS in N1, N3, and REM sleep%. The increase in the N1 sleep and decrease in the N3 and REM sleep in the nocturia group were likely to be the result of arousal due to nocturia. Although there was no difference in the AHI between the two groups, the increase in WASO and the decrease in sleep efficiency suggest that the sleep disturbance that prevents deep sleep was more severe in the nocturia group than in the non-nocturia group. This result is closely related to sleep quality and emotional disorders, and the results of sleep questionnaires, such as the PSQI-K and K-BDI-II, support this [25].

In the present study, female participants with OSAS were older than male participants with OSAS (50.1 vs. 59.4, *p*<0.001) and had a higher frequency of nocturia (35.8% vs. 59.8%, *p*<0.001). The AHI was lower in female participants with OSAS than in male participants with OSAS (34.8 vs. 24.6, *p*<0.001), and the ODI (29.3 vs. 21.0, *p*<0.001) and 90% ODI (11.5 vs. 6.2, *p*<0.001) were also lower in female participants with OSAS than in male participants with OSAS. Age is a well-known risk factor for nocturia [5]. We assumed that age-related voiding dysfunction and urinary disease may overpower the influence of OSAS on nocturia in female participants with OSAS. Therefore, in this study, the lack of association between nocturia and hypoxia-related parameters in female participants with OSAS may have been attributed to the characteristic of the age distribution and OSAS severity of the female group in this study. Furthermore, in 2020, Zhou et al.’s meta-analysis study reported that only men with OSAS were significantly associated with nocturia, showing a common finding with this study [22]. This also suggests that the present study’s result may be because the effect of OSAS on nocturia differs according to sex.

In 2009, Chung et al. reported that the mean oxygen saturation, 3% ODI, and 4% ODI, which are indices related to hypoxia on PSG, were variables that significantly affected OSAS-related nocturia in 304 patients, which is similar to our study’s findings [26]. However, the interpretation of other clinical factors known to affect nocturia (cardiovascular disease, kidney disease, use of diuretics, etc.) has not been accurately investigated, so the interpretation is limited.

In 2021, Hamada et al. conducted a study targeting 7,151 community residents and reported that the severity of OSAS assessed by 3% ODI was associated with the degree of nocturia [27]. This study also suggested that the severity of OSAS and severity of hypoxia are related to nocturia. However, there is a limitation in interpretation in terms of the accuracy of the evaluation, as it included pulse oximetry, actigraphy, and a questionnaire administered at the participant’s home; thus, PSG was not performed in a hospital.

The limitations of this study are as follows. First, the causal relationship between nocturia and related indicators could not be revealed because of the limitations of the cross-sectional study design. Second, we did not measure the levels of hormones (atrial natriuretic peptide, brain natriuretic peptide, and anti-diuretic hormone) known to be involved in the nocturia mechanism [28]. Third, a questionnaire on lower urinary tract symptoms, volume of nocturnal and daytime urine, or an evaluation of bladder function during the day was not conducted [29, 30]. Further studies are needed to investigate changes in hypoxia-related parameters and frequency of nocturia after treatment for OSAS such as positive airway pressure treatment or barbed reposition pharyngoplasty [31, 32]. Lastly, we did not review patients’ history of another treatment options for reducing nocturnal urine output such as behavior modification and off-label use of timed diuretics and desmopressin. It is reported that the use of a new formulation of desmopressin approved by the Food and Drug Administration, desmopressin acetate nasal spray appeared to benefit subset of patients with nocturia by a link between nasal function and chronic inflammatory state, also characteristic of OSAS [33, 34].

In conclusion, considerable number of patients with OSAS had nocturia and poor sleep quality. Based on this study’s results, male OSAS patients with severe hypoxia during sleep should be evaluated for nocturia.

## Supporting information

S1 Dataset (XLSX)
